# Age- and Sex-Specific Association Between Vegetation Cover and Mental Health Disorders: Bayesian Spatial Study

**DOI:** 10.2196/34782

**Published:** 2022-07-28

**Authors:** Abu Yousuf Md Abdullah, Jane Law, Christopher M Perlman, Zahid A Butt

**Affiliations:** 1 School of Planning University of Waterloo Waterloo, ON Canada; 2 School of Public Health Sciences University of Waterloo Waterloo, ON Canada

**Keywords:** mental health disorders, vegetation cover, age- and sex- specific association, Enhanced Vegetation Index, Bayesian, spatial, hierarchical modeling, marginalization, latent covariates

## Abstract

**Background:**

Despite growing evidence that reduced vegetation cover could be a putative risk factor for mental health disorders, the age- and the sex-specific association between vegetation and mental health disorder cases in urban areas is poorly understood. However, with rapid urbanization across the globe, there is an urgent need to study this association and understand the potential impact of vegetation loss on the mental well-being of urban residents.

**Objective:**

This study aims to analyze the spatial association between vegetation cover and the age- and sex-stratified mental health disorder cases in the neighborhoods of Toronto, Canada.

**Methods:**

We used remote sensing to detect urban vegetation and Bayesian spatial hierarchical modeling to analyze the relationship between vegetation cover and mental health disorder cases. Specifically, an Enhanced Vegetation Index was used to detect urban vegetation, and Bayesian Poisson lognormal models were implemented to study the association between vegetation and mental health disorder cases of males and females in the 0-19, 20-44, 45-64, and ≥65 years age groups, after controlling for marginalization and unmeasured (latent) spatial and nonspatial covariates at the neighborhood level.

**Results:**

The results suggest that even after adjusting for marginalization, there were significant age- and sex-specific effects of vegetation on the prevalence of mental health disorders in Toronto. Mental health disorders were negatively associated with the vegetation cover for males aged 0-19 years (−7.009; 95% CI −13.130 to −0.980) and for both males (−4.544; 95% CI −8.224 to −0.895) and females (−3.513; 95% CI −6.289 to −0.681) aged 20-44 years. However, for older adults in the 45-64 and ≥65 years age groups, only the marginalization covariates were significantly associated with mental health disorder cases. In addition, a substantial influence of the unmeasured (latent) and spatially structured covariates was detected in each model (relative contributions>0.7), suggesting that the variations in area-specific relative risk were mainly spatial in nature.

**Conclusions:**

As significant and negative associations between vegetation and mental health disorder cases were found for young males and females, investments in urban greenery can help reduce the future burden of mental health disorders in Canada. The findings highlight the urgent need to understand the age-sex dynamics of the interaction between surrounding vegetation and urban dwellers and its subsequent impact on mental well-being.

## Introduction

The relationship between vegetation and mental health remains an issue of considerable interest. The worldwide increase in urbanization has caused some unique environmental problems [1], such as substantial loss of vegetation-covered areas [2,3], which might adversely affect the mental health of urban dwellers. However, as mental health disorders can be of different categories and types [4,5], it is quite challenging to understand how adversely vegetation loss could affect the mental health conditions of the general public. Nevertheless, with increasing evidence that urban residents are particularly susceptible to various mental health disorders [6-9], it has become urgent to understand how vegetation could affect individuals of different ages and sex groups in an urban population.

Previous studies reported that vegetation could positively affect individuals with various mental health disorders, such as affective and psychotic disorders [10,11]. For example, in forest therapies, where people with affective disorders were involved in recreational activities in the nearest suburban forest, positive outcomes were reported, such as a reduction in symptoms of the disorders [11]. In addition to its utility in therapeutic intervention, exposure to an adequately managed vegetation-covered area, where people can tangibly and regularly experience the surrounding greenness, may significantly affect the psychology of people [12,13]. Unfortunately, because of increased urbanization, which mainly promotes the growth of commercial and residential areas, little attention has been paid to managing the already declining vegetation-covered areas.

As we consider age and sex differences in the prevalence of mental health conditions [14-17], we need to consider age and sex differences in susceptibility to reduced vegetation cover. The findings from previous studies suggest that the surrounding residential vegetation or greenness could be positively associated with better mental health outcomes for males aged younger than 65 years [18] and for young to middle-aged males (aged 30-45 years) and older females (aged ≥45 years) [19]. However, contrasting results are also present, where no age- and sex-specific differences in the association between the mental health conditions and surrounding greenness could be found for any of the study participants [20]. Therefore, the findings of previous studies are inconclusive and differ considerably from one another, indicating a need for further research.

Social inequality (or marginalization) can also contribute to poor mental health outcomes in urban areas [13,21] and complicate our understanding of the relationship between reduced vegetation and mental health disorders. Along with vegetation loss, social inequality has been identified as one of the major problems of urbanization. For example, rapid urbanization in China was accompanied by the rising marginalization of disadvantaged social groups such as laid-off workers and rural migrants [22]. In addition, highly urbanized census metropolitan areas in Ontario, Canada, with higher levels of marginalization, reported higher levels of psychotic disorders [21]. Hence, proponents of the concept that there is no significant association between reduced vegetation and poor mental health outcomes argue that people living in parts of urban areas with reduced vegetation cover could already be socioeconomically disadvantaged. Hence, they argue that the level of marginalization in urban areas could be a more important determinant of mental well-being than reduced greenery in these areas. Therefore, further understanding is warranted on whether vegetation’s age- and sex-specific effects on mental health could be significant after accounting for the influence of marginalization.

It is also important to note that the inconclusive findings of previous studies could be the outcomes of limitations in conventional analytical techniques. In this regard, special attention is required when selecting an appropriate vegetation measure to analyze the association between vegetation and common mental health disorders [1]. The vegetation measures that can accurately capture people’s exposure to surrounding greenness and adjust for environmental perturbations in urban areas are found to be best suited for population-based mental health studies [1,13]. In addition, spatial dependencies in the observed cases because of the influence of cases from neighboring regions need to be addressed to obtain unbiased estimates of the uncertainties and magnitude of the association between vegetation and mental health indicators [23,24]. Thus, a spatial modeling approach is required to adjust for any spurious spatial effects, such as spatial autocorrelation, and to account for any unmeasured spatial covariates that could influence the distribution of the observed cases [25].

Despite the modeling complexities and challenges, there is a need to understand the dynamics of the age- and sex-specific association between vegetation cover and mental health disorders. The importance of such a study stems from several critical issues. First, the demographic conditions in an area change over time, thus changing the mental health burden and the country’s mental health care costs [26]. Therefore, to understand the present and future mental health burdens, it is important to understand how a putative risk factor, such as reduced vegetation cover, can affect males and females in different age groups. Second, it is essential to understand which age and sex group could be particularly vulnerable to reduced vegetation cover for devising targeted intervention strategies [13,18,27]. Mental health promotion activities may differ based on the age and sex of the vulnerable groups [28]; therefore, it is imperative to identify the vulnerable groups to devise an effective intervention strategy. Third, as most studies attempt to understand the effect of people’s exposure to vegetation cover at the population level [1,13], the age- and sex-specific effects on mental health could be diluted because of grouping mental health disorder data into a single age and sex group. This may cause any adverse impact of reduced vegetation on different age and sex groups to remain undetected, thus silently creating a mental health disorder epidemic.

This detailed study extends a previous study on Toronto neighborhoods, which demonstrated the positive effects of vegetation cover on psychotic and non-psychotic disorders of both sexes (males and females) in the >0 year age group [1]. However, as the previous study mainly focused on the methodological aspects of selecting an appropriate vegetation measure to study the relationship with mental health disorders, it could not explore the relationship between vegetation cover and age- and sex-specific mental health disorder cases. Therefore, this cross-sectional study attempted to fulfill the existing research gap and aimed to understand the effect of vegetation cover on the mental well-being of males and females in 4 different age groups in Toronto, Canada. Specifically, this study aimed to examine the spatial association between vegetation cover and the age- and the sex-specific mental health disorder cases in Toronto neighborhoods after controlling for marginalization and unmeasured spatial and nonspatial covariates.

## Methods

### Study Area

The City of Toronto was selected as the study area because of its high urbanization rate, which led to a substantial increase in the built environment and loss of vegetation-covered areas [29]. All analyses in this study were conducted at the neighborhood level, which are geographic units created for planning and service delivery purposes by the Social Development and Administration Division of the City of Toronto. These units were constructed by aggregating the Statistics Canada census tracts into meaningful spatial units [1,30]. In 2016, there were 140 neighborhoods in Toronto with a population of 2,731,571 [31].

### Mental Health Disorder Data

Mental health disorder data were collected from the Ontario Community Health Profiles Partnership database [32]. The data, dated from April 1, 2015, to March 31, 2016 (fiscal year 2015), was generated from the study, *Enrollment, Access, Continuity, and Mental Health Gaps in Care (Institute for Clinical Evaluative Sciences Project No. 2018 0900 992 000).* This study was supported by the Institute for Clinical Evaluative Sciences, a nonprofit corporation funded by the Ontario Ministry of Health and Long-Term Care. Further details on the project, the primary data sources, and the inclusion and exclusion criteria for generating the data set can be found in the project report [6].

**Figure 1:**
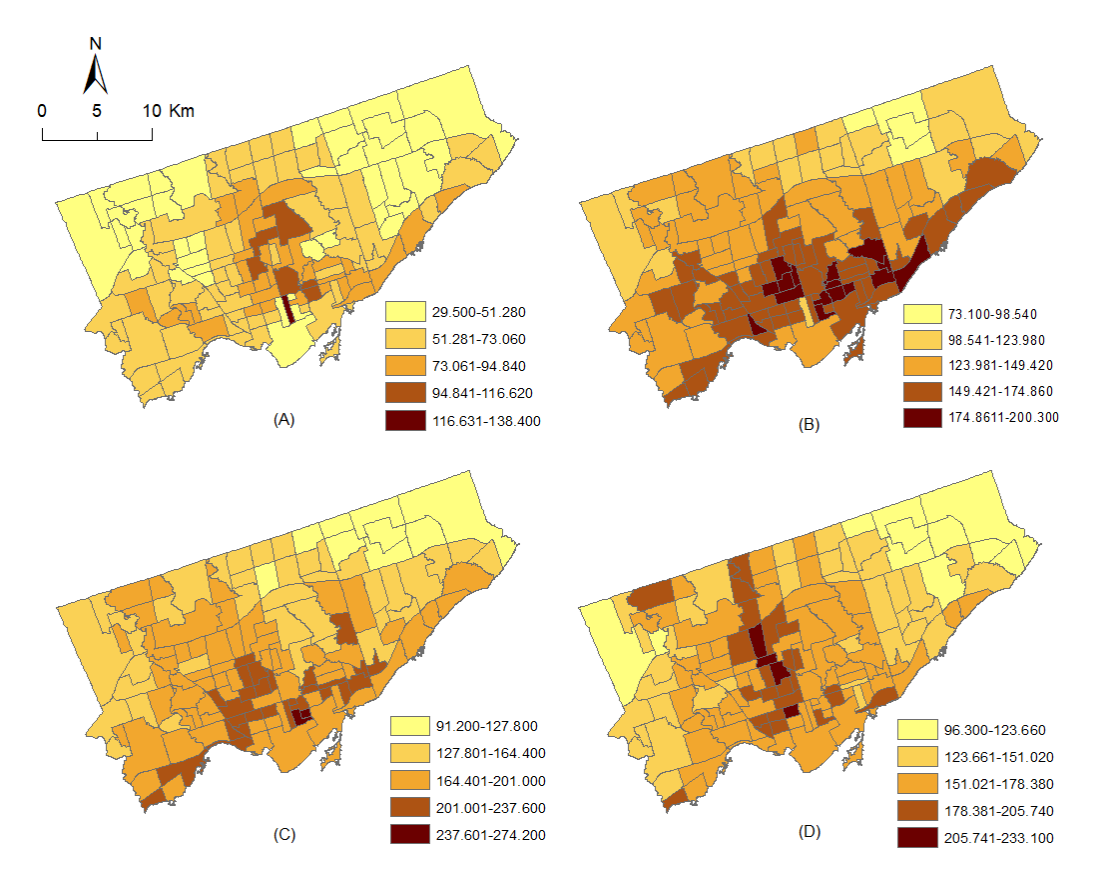
The crude rates (per 1000 population) of the combined mental health disorders for both sexes in the (A) 0-19, (B) 20-44, (C) 45-64, and (D) ≥65 years age groups in the Toronto neighborhoods.

The retrieved data set comprised 4 major mental health disorders (Table 1), each created from various subcategories defined by the Ontario Health Insurance Plan codes. The combined mental health disorder category in the data set was created by adding the counts of these 4 major categories. The age- and sex-stratified observed counts for the combined mental health disorder variable were used in this study. Further details on the 4 major categories and their subcategories are provided in Table 1. This study used the combined mental health disorder data for 4 age groups: 0-19, 20-44, 45-64, and ≥65 years. Figure 1 shows the crude rates of the combined mental health disorders for males and females in the 4 age groups and the spatial distribution of the disorders in the study area.

Figure 1 shows that the mental health disorders in Toronto exhibit considerable spatial variations. For example, the rate of mental health disorders among individuals aged 20-44 years is the highest in Toronto’s south-central parts. Similarly, for the remaining age groups, the rates were generally higher in the southern parts than in the other areas. Therefore, to measure the spatial autocorrelation in the retrieved data, global Moran’s I tests were performed using the GeoDa software [33]. The results suggested moderate autocorrelation (Moran’s I=0.491; *P*=.001) in the data for the age group 0-19 years and high autocorrelation (Moran’s I=0.702, *P*=.001; Moran’s I=0.717, *P*=.001; and Moran’s I=0.696, *P*=.001, respectively) for the 20-44, 45-64, and ≥65 years age groups. Hence, significant spatial autocorrelation in the data necessitated selecting a spatial modeling technique for this study.

**Table 1:** The 4 major categories and subcategories of mental health disorders with Ontario Health Insurance Plan (OHIP) codes.

Major category and subcategory		OHIP code (subcategory)
**Psychotic disorders**		
	Schizophrenia	295
	Manic-depressive psychoses and involutional melancholia	296
	Other paranoid states	297
	Other psychoses	298
**Non-psychotic disorders**		
	Hysteria, reactive depression, neurasthenia, obsessive-compulsive neurosis, and anxiety neurosis	300
	Personality disorders	301
	Sexual deviations	302
	Psychosomatic illness	306
	Adjustment reaction	309
	Depressive disorder	311
**Substance-use disorders**		
	Alcoholism	303
	Drug dependence	304
**Family, social, and occupational issues**		
	Economic problems	897
	Marital difficulties	898
	Parent-child problems	899
	Problems with aged parents or in-laws	900
	Family disruption or divorce	901
	Education problems	902
	Social maladjustment	904
	Occupational problems	905
	Legal problems	906
	Other problems of social adjustment	909
**Combined mental health disorders**		
	Psychotic, non-psychotic, substance-use and family, social and occupational issues related disorders	All codes listed above

### Vegetation Cover Data

The vegetation cover, specifically the density and quality (biomass vigor) of vegetation, was estimated using remote sensing techniques. For this purpose, 3 satellite images from the Landsat Operational Land Imager and Thermal Infrared Sensor or Landsat 8 were obtained from the United States Geological Survey EarthExplorer data repository [34]. The downloaded images had a spatial resolution of 30 m; contained an average cloud cover of 2.67%; and were captured between May 20, 2016, and June 14, 2016. Thus, the vegetation cover during the spring-summer seasons in 2016 was measured to be consistent with the data period of the mental health disorder data set. After radiometric and atmospheric correction, the images were mosaiced and clipped to the extent of the study area.

In this study, the Enhanced Vegetation Index (EVI) was calculated to estimate the vegetation cover in the Toronto area [35]. The EVI was calculated from the processed satellite image and using the *Raster Calculator* function in ArcMap 10.7 software [36] from Environmental Systems Research Institute. Vegetation indexes, such as EVI, are calculated by measuring the relative abundance of different electromagnetic waves (commonly known as bands) reflected from the surface of the vegetation to the sensors in the satellite. The details of the index and the formula used to calculate EVI can be found in Multimedia Appendix 1 [1,35].

### The Bayesian Spatial Hierarchical Modeling

The age- and sex-stratified associations between vegetation and mental health disorders were analyzed using the Bayesian spatial hierarchical modeling technique. In the Bayesian spatial hierarchical models, the observed counts, *O_i_,* of the combined mental health disorder in each neighborhood *i* (where i=1,2,…140) were assumed to follow a Poisson distribution *(O_i_ ~ Poisson(λ_i_))*. Here, *λ_i_* denotes the expected number of mental health disorder cases in the neighborhood *i*.

Furthermore, the observed count of the combined mental health disorders in a neighborhood (*i*) was assumed to be a function of the unknown area-specific relative risk of the disorders, *r_i_*, and the expected count, *E_i_*. The expected counts of the combined mental health disorders were calculated separately for males and females in each age group. Specifically, the expected counts were calculated by multiplying the age- and sex-specific rates of the disorders with the residential population of each neighborhood [25]. The age- and sex-specific rates of the disorder were calculated by dividing the total number of mental health disorder cases in each age and sex group by the total number of individuals (population) in that age and sex group of the neighborhood. Thus, through the integration of *E_i_*, the population at risk was accounted for in the Bayesian spatial hierarchical models.

In addition, the environmental and socioeconomic risk factors, such as reduced vegetation cover and marginalization, in a neighborhood could be assumed to govern the risk of developing different mental health disorders [1,8,11,25,37,38]. Therefore, the unknown area-specific relative risk in a neighborhood was modeled as a function of the vegetation cover and the marginalization factors. For this study, we mainly focused on modeling the spatial association between the age- and sex-stratified mental health disorder cases and the amount of vegetation cover in each neighborhood (*X_1i_*). However, to adjust for social inequality or marginalization, we also added the 4 domains (or variables) of marginalization in the Bayesian spatial hierarchical models: material deprivation (*X_2i_*), ethnic concentration (*X_3i_*), residential instability (*X_4i_*), and dependency (*X_5i_*) covariates. All the domains were retrieved from the Ontario Marginalization Index (OMI) [39], and the weighted average score for each variable was used as a measure of the covariate. Before including the 4 OMI variables in the models, we carried out Pearson correlation and multicollinearity tests [40] and confirmed the absence of any multicollinearity among the variables. A detailed explanation for selecting these 4 socioeconomic covariates and the results of the multicollinearity tests can be found in our previous study [1]. The 4 OMI variables are described more elaborately elsewhere [39].

Hence, the observed counts of the combined mental health disorders in any neighborhood can be modeled as:



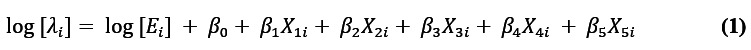



However, because of nonspatial variations in individual-level processes or variations in individual-level risks of developing mental health disorders, overdispersion could be considered a common problem for count data 
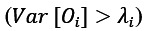
 [25,41]. In addition, spatial autocorrelation could be present in spatial data because of unmeasured (latent) spatial covariates influencing the distribution of the observed variable (here, the mental health disorder cases). As previously mentioned in the Mental Health Disorder Data section, we found moderate to high spatial autocorrelation in our mental health disorder data set. Therefore, to adjust for the nonspatial and spatially structured unknown covariates, we integrated 2 Gaussian random-effects terms, *u_i_* and *s_i_*, using equation 1 to obtain the final model equation [25]:



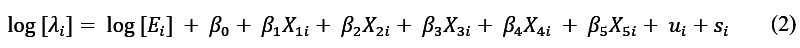



Here, the terms *u_i_* and *s_i_* represent all the covariates (nonspatial and spatial, respectively) that we could not measure but could have influenced the distribution of the mental health disorders in the study area.

We also analyzed whether the nonspatial or spatially structured unmeasured covariates had significantly influenced the Bayesian models. Their relative contributions were modeled using the posterior distribution of the quantity *ψ* and could be defined as follows [42]:



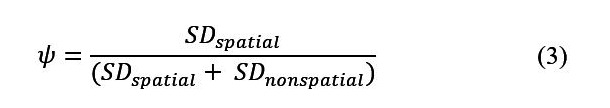



where SD_spatial_ is the empirical marginal SD of s*_i_* and SD_nonspatial_ is the empirical marginal standard deviation of *u_i_*.

On the basis of equation 3, as *ψ*→1, the variations in the area-specific relative risk of mental health disorders would be mainly spatial, and as *ψ*→0, the variations would be nonspatial in nature.

Once the required models were run, we analyzed the relative risk of developing mental health disorders because of reduced vegetation cover in each neighborhood. The relative risk of mental health disorders for the 4 age groups of males and females in the neighborhood *i* can be expressed as follows:



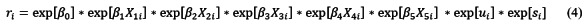



### Ethics Approval

No ethics approval was required for this work. All data used for the analysis and reporting of the results are in concordance to the free usage and sharing policy guidelines provided by the data providers.

## Results

### The Bayesian Spatial Hierarchical Modeling: The Age- and Sex-Stratified Association Between EVI and Mental Health Disorder Cases

The results of the age- and sex-stratified spatial association between vegetation cover (EVI) and mental health disorder cases at the neighborhood level are tabulated in Table 2. The results suggest that the mental health of males in the 0-19 and 20-44 years age groups is significantly affected by the surrounding vegetation cover. By contrast, the association between urban greenery (represented by EVI) and mental health was significant only among females aged 20-44 years. For males in the 0-19 and 20-44 years age groups, the magnitude of the association between vegetation and mental health disorders was *β*_1_=−7.009 (95% CI −13.130 to −0.980) and *β*_1_=−4.544 (95% CI −8.224 to −0.895), respectively. However, for females in the 20-44 years age group, this magnitude was *β*_1_=−3.513 (95% CI −6.289 to −0.681). Hence, the results indicate that increased vegetation cover could potentially improve the mental health conditions of both males and females in their youth. In particular, males from the 0-19 years age group could most benefit from the presence of vegetation.

The *ψ* values for all the models are >0.70 and are statistically significant. As all the *ψ* values are approaching 1 (*ψ*→1), the variations in the area-specific relative risk of mental health disorders for both sexes and all age groups could be predominantly influenced by the unmeasured spatial covariates.

**Table 2:** The results of analyzing the spatial association between Enhanced Vegetation Index (EVI) and combined mental health disorders of males and females in 4 different age groups (years).

Summaries of the posterior means		0-19 years age group	20-44 years age group	45-64 years age group	≥65 years age group
**Men**					
	*β*_0_ (95% CI)	*0.550 (0.232 to 0.873)^a^*	*0.274 (0.078 to 0.472)*	0.138 (−0.056 to 0.333)	*0.221 (0.004 to 0.438)*
	Vegetation cover/EVI *β*_1_ (95% CI)	−*7.009 (−13.130 to −0.980)*	−*4.544 (−8.224 to −0.895)*	−2.920 (−6.585 to 0.721)	−3.841 (−7.913 to 0.166)
	Material deprivation, *β*_2_ (95% CI)	−0.035 (−0.097 to 0.027)	*0.095 (0.056 to 0.134)*	*0.132 (0.093 to 0.169)*	*0.053 (0.012 to 0.093)*
	Ethnic concentration, *β*_3_ (95% CI)	−*0.192 (−0.265 to −0.121)*	−*0.148 (−0.194 to −0.102)*	−*0.156 (−0.201 to −0.110)*	−*0.079 (−0.127 to −0.030)*
	Residential instability, *β*_4_ (95% CI)	−0.013 (−0.073 to 0.046)	*0.080 (0.040 to 0.119)*	*0.136 (0.098 to 0.174)*	*0.075 (0.035 to 0.116)*
	Dependency, *β*_5_ (95% CI)	−*0.131 (−0.228 to −0.034)*	−*0.074 (−0.137 to −0.012)*	−*0.109 (−0.174 to −0.044)*	−0.022 (−0.086 to 0.043)
	*ψ*^b^ (95% CI)	*0.716 (0.520 to 0.899)*	*0.795 (0.647 to 0.904)*	*0.742 (0.558 to 0.886)*	*0.817 (0.688 to 0.908)*
**Women**					
	*β*_0_ (95% CI)	*0.325 (0.001 to 0.645)*	*0.292 (0.141 to 0.439)*	0.137 (−0.017 to 0.292)	*0.214 (0.015 to 0.413)*
	Vegetation cover/EVI, *β*_1_ (95% CI)	−1.624 (−7.632 to 4.448)	−*3.513 (−6.289 to −0.681)*	−1.376 (−4.274 to 1.533)	−2.934 (−6.660 to 0.784)
	Material deprivation, *β*_2_ (95% CI)	−0.029 (−0.089 to 0.032)	*0.096 (0.066 to 0.125)*	*0.093 (0.063 to 0.123)*	0.023 (−0.016 to 0.061)
	Ethnic concentration, *β*_3_ (95% CI)	−*0.262 (−0.334 to −0.192)*	−*0.186 (−0.221 to −0.151)*	−*0.144 (−0.180 to −0.108)*	−*0.104 (−0.150 to −0.058)*
	Residential instability, *β*_4_ (95% CI)	0.013 (−0.046 to 0.072)	*0.048 (0.019 to 0.078)*	*0.071 (0.041 to 0.101)*	*0.068 (0.030 to 0.107)*
	Dependency, *β*_5_ (95% CI)	−0.069 (−0.165 to 0.026)	−*0.068 (−0.115 to −0.021)*	−*0.064 (−0.112 to −0.016)*	0.034 (−0.025 to 0.092)
	*ψ*^b^ (95% CI)	*0.738 (0.544 to 0.906)*	*0.799 (0.675 to 0.892)*	*0.779 (0.632 to 0.887)*	*0.837 (0.729 to 0.914)*

^a^The statistically significant values at a 95% CI have been italicized.

^b^The relative contribution of the spatially structured and nonstructured random effect terms.

### The Variations in Relative Risks of Mental Health Disorders for Men and Women in Different Age Groups

**Figure 2:**
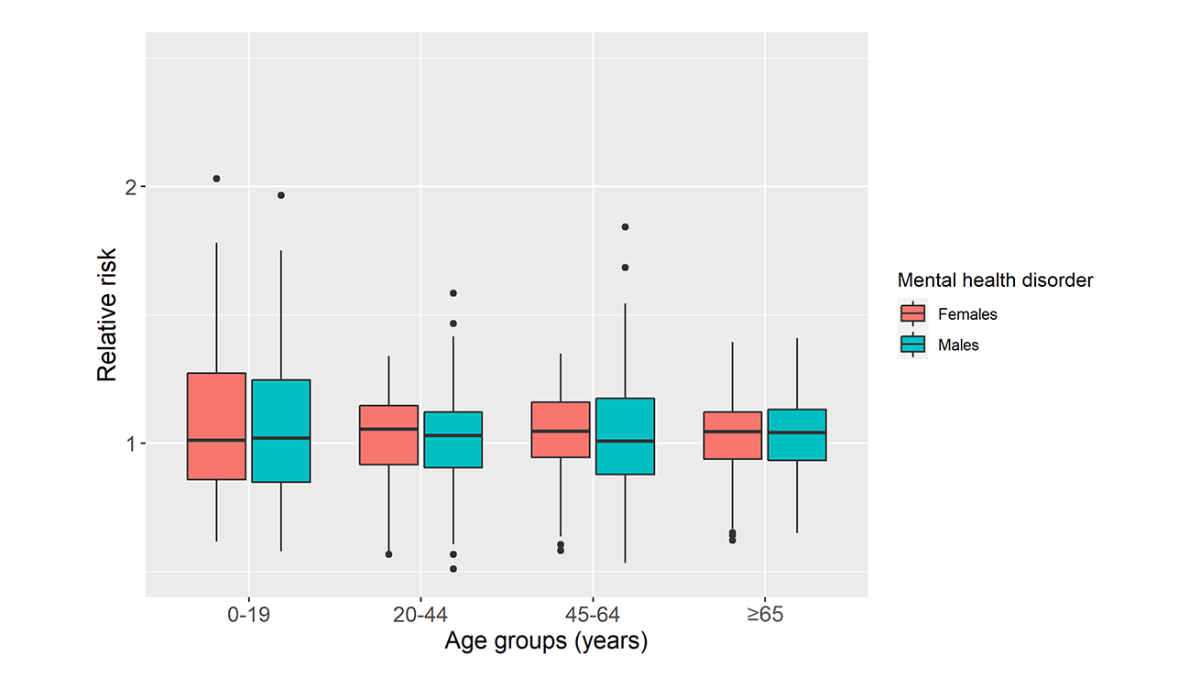
The posterior mean relative risks of combined mental health disorders for males and females in the 4 age groups (0-19, 20-44, 45-64, and ≥65 years).

The relative risk values of the combined mental health disorders for males and females in the 0-19, 20-44, 45-64, and ≥65 years age groups in the neighborhoods of Toronto are compared in Figure 2. The relative risks were calculated using equation 4. The results suggest similarities in relative risk values for males and females aged 0-19 and ≥65 years. However, females had higher relative risks for the 20-44 and 45-64 years age groups than males in the same age groups. All median values of relative risks were >1, implying an increased risk of developing various mental health disorders. Although males in the 20-44 and ≥65 years age groups were particularly vulnerable, females in the 20-44 and 45-64 years age groups experienced the highest risk.

**Figure 3:**
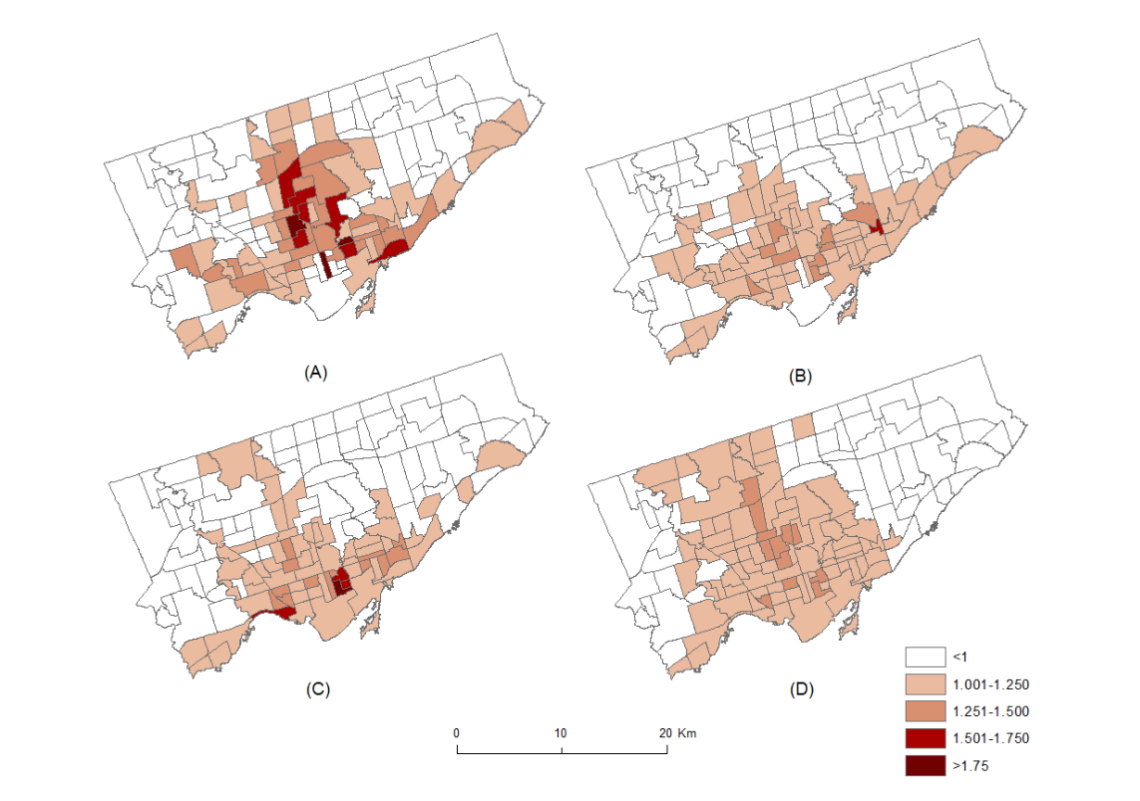
The posterior mean of relative risk (r_i_) for males in the (A) 0-19, (B) 20-44, (C) 45-64, and (D) ≥65 years age groups.

**Figure 4:**
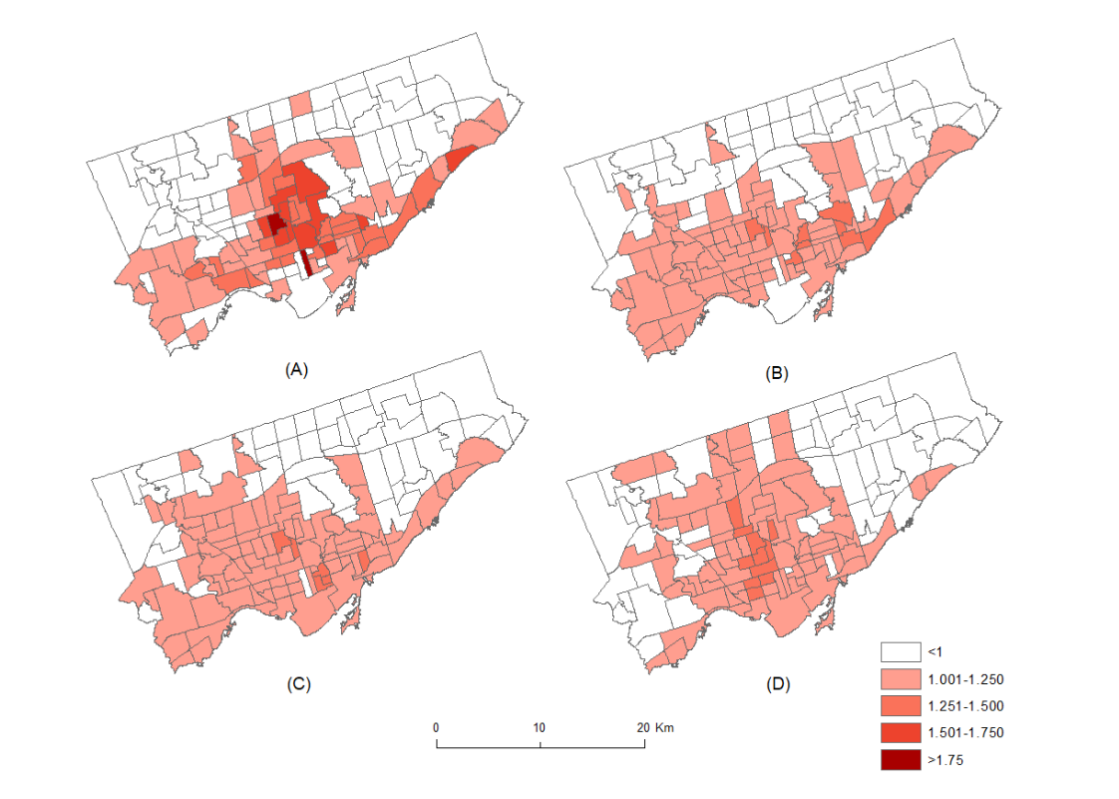
The posterior mean of relative risk (r_i_) for females in the (A) 0-19, (B) 20-44, (C) 45-64, and (D) ≥65 years age groups.

The combined mental health disorder risks because of varying vegetation cover in each neighborhood after adjusting for the risks from marginalization and unmeasured covariates are shown in Figures 3 and 4. The relative risk maps were similar for males and females in all age groups, suggesting an identical risk distribution for both sexes. However, a comparison of the maps of different age groups for each sex revealed interesting distribution patterns. For example, Figures 3A and 4A for males and females in the 0-19 years age group, respectively, showed that the neighborhoods with high risk (^r^*i*>1) were located in the central Toronto area. By contrast, Figures 3B and 4B show that high-risk areas for the 20-44 years age group for both sexes were located in the southern parts of the study area. Although high-risk neighborhoods in the Figures 3C and 4C for the 45-64 years age group of both males and females were localized in the southern parts, Figure 4C illustrates that the risk for females was distributed over a larger area compared with the risk for males. A good portion of the central western part of Toronto was at an elevated risk of mental health disorders for females in the 45-64 years age group. In contrast, high-risk neighborhoods in Figures 3D and 4D for males and females aged ≥65 years were found in the south to northward directions in the study area.

## Discussion

### Principal Findings

Through the application of Bayesian spatial hierarchical modeling and remote sensing techniques, this study found that reduced vegetation cover was significantly associated with poor mental health outcomes in young males and females in urban Toronto. The relative risk maps identified hotspots of mental health disorder cases, which could be targeted for future interventions. Although the mental health disorder cases for young males (aged 0-19 and 20-44 years) showed a higher magnitude of association with reduced vegetation cover than females, the absolute values of the relative risk of mental health disorder cases were higher for adult females (aged 20-44 and 45-64 years). These findings reflect the complexities of studying the age- and sex-specific association between vegetation cover and mental health disorders. The results also highlight the importance of further research to understand the exact dynamics between the surrounding greenery and mental health disorders in urban areas.

The results suggest that vegetation was associated with mental health disorder cases in Toronto for females in the 20-44 years age group and males in the 0-19 and 20-44 years age group. However, this study could not detect any significant association between vegetation and mental health disorders for the middle-aged (45-64 years) and older adults (≥65 years). These findings are concordant with the results from previous studies on people from similar age groups but in different study settings. For example, higher surrounding greenness was found to be associated with improved neurobehavioral health in children [43]. In addition, the research suggested that improved mental health conditions such as improved attention, reduced symptoms of aggressive behavior, and also, reduced symptoms of attention deficit hyperactivity disorder were evident in children aged 6-18 years because of an increase in surrounding greenness. The associations were significantly prominent for the externalizing behaviors, especially when the greenness was within 1600 m of the children’s residence. Similarly, another review study concluded that neighborhood greenness was beneficial for children’s cognitive function and mental health [44].

As evidenced in our results, exposure to urban greenery could have a more prominent effect on young adults aged 20-44 years than older adults. This differential effect could be explained by reflecting on how people in different age groups engage in social cohesion and health beneficial activities. Young people are relatively more socially engaging, physically active, and more likely to maintain health-benefiting behaviors than older adults [45]. Consequently, owing to more outdoor activities than older adults, young people could be exposed to greater levels of urban greenery. Furthermore, previous research reported that the utility of greenspace lies not only as a site of physical activities but also in its restorative effect on mental health [13]. Hence, young adults may actively seek vegetation-covered areas and other forms of urban greenery because of their utility as sites for physical exercise and destressing [27,46]. Moreover, various types of neighborhood vegetation, such as trees, can lead to greater outdoor space use and increased social engagements among youth [47-49]. These social engagements, in turn, have the potential to promote social cohesion and mental well-being [13,27].

A crucial benefit of using EVI in this research to model the association between vegetation cover and the age- and sex- stratified mental health disorder cases could be discussed using findings from previous studies. Several prominent studies have discussed the importance of using vegetation measures that can capture both the quality and quantity of the surrounding greenery [1,50,51]. For example, a study conducted in Western Australia strongly emphasized the quality rather than the quantity (or number) of green spaces in reducing the psychological distress of people [50]. In concordance with these studies, vegetation indexes were recommended for use in mental health studies instead of the conventional areal-based measures of vegetation [1]. In this regard, sophisticated indexes such as EVI could be particularly suitable because they are highly sensitive to the leaf structures and photosynthetic activities in the vegetation patches. In addition, vegetation indexes such as EVI can also adjust for various disturbances such as canopy background noise and atmospheric resistance, which can greatly improve the detection of different types of vegetation in an urban setting.

The findings from this study provided critical evidence that reduced vegetation cover can impair the mental health of young people. Hence, this study demonstrated that based on the demographics (age and sex structures) in an area, investment in vegetation could help reduce the burden of mental health disorders. In addition, the results suggest a need to assess why, unlike the males in the 0-19 years age group, the mental health conditions of children and adolescent females are not affected by reduced vegetation cover. Previous studies have shown that the conservative approaches of societies toward females might cause young females to spend less time outside their homes [52,53], leading to less exposure to surrounding vegetation and thus possibly reducing the influence of vegetation on their mental health. Future studies can attempt to understand the relative exposure of young females to vegetation-covered surroundings by measuring their outdoor activities. Finally, although no association between mental health disorders in older adults (aged 45-64 and ≥65 years) and vegetation cover was found in this study, urban residents from these age groups should still be encouraged to spend time in vegetation-covered surroundings, as the present evidence suggests that such activity could lead to improved mental health outcomes [1,13,54].

Our study is a spatial cross-sectional study that demonstrates how the vegetation cover in an urban landscape could be potentially associated with mental health disorder cases in Toronto. It captures how the observed distribution of cases in the specific study period had resulted from the continuous influence of the risk factors in previous years. Various socioeconomic and environmental factors influence mental health disorder cases, with distinctive effects on males and females in different age groups [1,13,38,55]. However, these risk factors are required to function continuously in an area (continued exposure) to significantly affect the cases in a large area such as the City of Toronto. Therefore, although we could not incorporate temporal analyses owing to the unavailability of mental health spatial data in different years, the findings still reflect the outcome of changes in vegetation in the preceding years and how this change could have affected the cases in the study area. Thus, the most significant contribution of this study lies in advancing the knowledge on reduced vegetation cover and how it could be a risk factor for the mental health disorders of males and females in different age groups in an urban area. There is a paucity of conclusive evidence in the existing literature, which this research has attempted to fulfill.

Despite the strengths, several limitations should be considered when interpreting the results of this study. First, as this was an ecological study, no individual-level conclusions should be deduced from the results. The results are only applicable to groups of people at the neighborhood level in Toronto. Second, the combined mental health disorder data set, which was used as the outcome variable, was created by aggregating different subcategories of mental health disorders. Better results could be obtained if the age- and sex-specific associations were analyzed using each subcategory that created the combined data set. However, as the original data set did not contain the observed cases for each subcategory, the combined data set had to be used. Third, this study analyzed the association between mental health disorder cases and vegetation cover in the summer-spring period. In reality, there could be a seasonal effect on the association because people’s activities may change during a bitterly cold winter and also because people are more prone to develop seasonal affective disorders during seasons such as fall and winter. In this regard, the 2 spatial and nonspatial random effect terms in the Bayesian models would have adjusted for any unmeasured effects in the associations. Fourth, while interpreting the results, it is important to consider that people already in better mental health conditions could be more socioeconomically advantaged and thus reside in cities rich with vegetation cover [56]. However, we considered this effect and source of potential bias during our analysis. On the basis of the findings of several prominent studies [13,21,22], we modeled this issue as a product of the socioeconomic construct in the study area. Consequently, we adjusted for social inequality using the 4 marginalization covariates, which potentially adjusted for the differential exposure to vegetation cover owing to socioeconomic status and residential location in vegetation-rich areas. Finally, the findings from this research could only be generalized to areas having similar physical and socioeconomic conditions like the City of Toronto.

Despite its limitations, this study focused on the much-debated area of mental health research and aimed to analyze in detail the age- and sex-specific association between vegetation cover and mental health disorders. This research attempted to overcome the complex spatial and nonspatial modeling constraints, which are generally overlooked in mental health studies. The findings from this study provide crucial evidence that reduced vegetation cover could disproportionately affect the mental health conditions of young people. Owing to increased urbanization across the globe, with significant loss of vegetation-covered areas, the burden of mental health disorders among youth is likely to increase. This highlights the need to reconsider urban planning strategies and develop greener and more sustainable cities for future generations.

### Conclusions

Despite strong evidence of the age- and sex-specific differences in the prevalence of mental health disorders, few studies have attempted to understand the age- and sex-specific association between vegetation cover and mental health disorders in urban areas. With the rapid loss of vegetation because of urbanization and global environmental changes, there is a need to thoroughly assess the impact of reduced vegetation on males and females from different age groups. Therefore, this study analyzed the association between vegetation cover and the combined mental health disorder cases for males and females in the 0-19, 20-44, 45-64, and ≥65 years age groups in the neighborhoods of Toronto. An EVI was constructed from Landsat-8 imageries using remote sensing techniques, and the Bayesian spatial hierarchical modeling was used to model the association between vegetation and mental health disorder cases after accounting for material deprivation, ethnic concentration, residential instability, dependence, and unmeasured covariates. The spatial and nonspatial unmeasured covariates were adjusted in the models by integrating 2 Gaussian random effect terms, which also helped to adjust for the spatial autocorrelation and overdispersion in the mental health disorder data. The age- and sex-specific analyses found that the mental health of children, adolescents, and younger adults could be particularly susceptible to reduced vegetation cover. Specifically, both males and females aged 0-19 and 25-44 years were vulnerable to various mental health disorders. In contrast, no significant association was evident between vegetation and the mental health disorder cases of adults in the 45-64 and ≥65 years age groups. For the older age groups, the socioeconomic factors were found to be more significantly influential than the variations in vegetation cover. The relative risk maps identified localized hotspots of mental health disorder cases, which could be the focus of urban vegetation management practices. The study findings suggest that the young urban population could be highly susceptible to reduced vegetation cover, which may lead to deterioration of mental health conditions in the early stages of life. The results highlight the need for sustainable urban planning initiatives that prioritize the conservation and growth of urban greenery. Although the study was conducted for the residents of Toronto, Canada, similar results could be expected in areas with comparable physical and socioeconomic conditions.

## Appendix

Multimedia Appendix 1 Calculation of the Enhanced Vegetation Index and implementation of the Bayesian Spatial Hierarchical Model.

## Abbreviations

EVI: Enhanced Vegetation Index
OMI: Ontario Marginalization Index
