# Supplementary material for: Age- and Sex-Specific Association Between Vegetation Cover and Mental Health Disorders: Bayesian Spatial Study
Source: JMIR Public Health Surveill. 2022 Jul 28;8(7):e34782. doi: 10.2196/34782 (PMC9377430; doi:10.2196/34782)
Supplement: Multimedia Appendix 1 [file publichealth_v8i7e34782_app1.docx]

**Appendix A: Details of the formula used to calculate the Enhanced Vegetation Index (EVI)**

The formula used to calculate the Enhanced Vegetation Index is defined by Equation 1 [1]:

|  | $\mathrm{EVI}= G * ((NIR - R) / (NIR + C1 * R - C2 * B + L_{\mathrm{EVI}}))$ | (1) |
| --- | --- | --- |
| where,  **NIR is the Near Infrared band of the satellite image**  **R is the Red band of the satellite image**  **B is the Blue band of the satellite image**  **G is the gain factor that makes EVI comparable to that of other vegetation indices**  **L_EVI_ is a constant used to adjust for the canopy background**  C1 and C2 are constants used to adjust for atmospheric resistance | | |

For Landsat 8, Equation 1 could be rewritten to give Equation 2 [1]:

|  | $\mathrm{EVI}= 2.5 * ((Band 5 - Band 4) / (Band 5 + 6 * Band 4 - 7.5 * Band 2 + 1))$ | (2) |
| --- | --- | --- |

Equations 1 and 2 show that EVI measures the vegetation content in an area using the relative amount of blue, red, and near-infrared waves reflected from a vegetation cover. Additionally, the canopy background noise and the atmospheric resistance are adjusted during the calculation of EVI. Higher values of EVI indicate dense and healthy vegetation content in an area. For a detailed justification of why the EVI was selected for measuring the vegetation cover in the study area, the readers are requested to consult Abdullah et al. (2021) [2].

**Appendix B: Details of the implementation process for the Bayesian spatial model**

We used the WinBUGS software [3] from The BUGS Project (Cambridge, London) to fit the models defined by Equation 4. A normal distribution with an expected mean of 0 and a precision (1/variance) of 0.00001 was defined as the prior for the slopes ($\beta_{1}$ to $\beta_{5}$), whereas an intrinsic conditional autoregressive (ICAR) distribution was specified for the random effect terms ($u_{i}$ and $s_{i}$). The precision parameters of the model were varied (within any neighborhood) using a Gamma $(Г)$ distribution (a,b) with a mean of $\frac{a}{b}$ and variance of $\frac{a}{b^{2}}$ . In this study, we employed the prior distribution of $Г$ (0.001, 0.001) for both $u_{i}$ and $s_{i}$ and assigned an improper uniform prior, dflat() for the intercept term, $\beta_{0}$ [2, 4].

Two chains having different initial values were run to achieve convergence to the targetted posterior distribution. We checked for convergence by visually inspecting the trace plots, the serial autocorrelation function, and the Gelman-Rubin diagnostic. Furthermore, we assessed the accuracy of the distribution using the Monte Carlo (MC) error of the posterior mean for each parameter. We only considered models that had fully converged and had MC error less than 5% of the sample (posterior) deviation.

**References**

1. USGS. Landsat Surface Reflectance-Derived Spectral Indices - Landsat Enhanced Vegetation Index. 2019 [December 20, 2019]; Available from: <https://www.usgs.gov/land-resources/nli/landsat/landsat-enhanced-vegetation-index?qt-science_support_page_related_con=0#qt-science_support_page_related_con>.

2. Abdullah AYM, Law J, Butt ZA, Perlman CM. Understanding the Differential Impact of Vegetation Measures on Modeling the Association between Vegetation and Psychotic and Non-Psychotic Disorders in Toronto, Canada. International Journal of Environmental Research and Public Health. 2021;18(9):4713.

3. Spiegelhalter D, Thomas A, Best N, Lunn D. WinBUGS user manual; 2003.

4. Law J, Haining R, Maheswaran R, Pearson T. Analyzing the relationship between smoking and coronary heart disease at the small area level: a Bayesian approach to spatial modeling. Geographical Analysis. 2006;38(2):140-59.
